# Digital era in oral rehabilitation: a scoping review of trends and implications for clinical practice

**DOI:** 10.1590/1807-3107bor-2026.vol40suppl1066

**Published:** 2026-08-21

**Authors:** Adriana da Fonte Porto CARREIRO, Anne Kaline Claudino RIBEIRO, Gülce ÇAKMAK, Walter Yu Hang LAM, Wei-Shao LIN, Ji-Man PARK, Murali SRINIVASAN, Manabu KANAZAWA, Amirali ZANDINEJAD, Burak YILMAZ

**Affiliations:** (a) Universidade Federal do Rio Grande do Norte - UFRN, Department of Dentistry, Natal, RN, Brazil; (b) Charité-Universitätsmedizin Berlin, Department of Prosthodontics, Geriatric Dentistry and Craniomandibular Disorders, Berlin, Germany; (c) University of Hong Kong, Faculty of Dentistry, Hong Kong Special Administrative Region, China; (d) Indiana University School of Dentistry, Department of Prosthodontics, Indianapolis, IN, USA; (e) Seoul National University, Department of Prosthodontics and Dental Research Institute, Seoul, Republic of Korea; (f) University of Zurich, Center of Dental Medicine, Clinic of General-, Special care- and Geriatric Dentistry, Zurich, Switzerland; (g) Institute of Science Tokyo, Graduate School of Medical and Dental Sciences, Gerodontology and Oral Rehabilitation Department, Tokyo, Japan; (h) University of Rochester, School of Medicine and Dentistry, Department of Prosthodontics, New York, USA; (i) University of Bern, School of Dentistry, Department of Gerodontology and Reconstructive Dentistry, Bern, Switzerland

**Keywords:** Computer-Aided Design, Denture, Complete, Dental Prosthesis, Implant-Supported, Photogrammetry, Scoping Review

## Abstract

This scoping review mapped the evidence on the clinical applicability and implications of digital workflows for fixed and removable full-arch rehabilitation in completely edentulous patients, focusing on workflow-related operational parameters and clinician-reported outcomes. This review was conducted in accordance with the PRISMA-ScR and was registered on the Open Science Framework. The research question was "What is the current evidence on digital workflows for full-arch rehabilitations and their implications for clinical practice?". A search was conducted in PubMed/MEDLINE, Web of Science, Scopus, Cochrane Library, and Embase, supplemented by grey literature and reference screening. The inclusion criteria were clinical studies and reviews focusing on the clinical implications of workflow-related performance indicators and the clinician's perceptions, considering the digital workflow. A descriptive analysis was conducted in terms of clinical application and key findings regarding accuracy, time-efficiency, cost-effectiveness, and clinician satisfaction. A total of 40 studies were included. The implant-level impressions demonstrated values for 3D linear deviations within clinically acceptable thresholds (up to 200 μm) and traditional methods remained feasible for tissue impressions due to the highest discrepancies found in the peripheral regions. The digital workflow was time- and cost-saving, ranging from 27.17% to 75.10% and 9.21% to 39.49%, respectively. The digital workflow was accurate for implant-level impressions. Evidence suggests limitations in peripheral and movable mucosa capture; however, certain protocols may improve reliability, and conventional methods remain important in specific clinical contexts. Moreover, it's time- and cost-efficient for interventions in completely edentulous patients, but the evidence remains scarce and inconclusive for clinician satisfaction.

## Introduction

The conventional workflow for full-arch implant rehabilitation is labor-intensive and highly technique-sensitive, requiring substantial time and professional expertise. Over the last years, groundbreaking advancements in computer-aided design and computer-aided manufacturing (CAD-CAM) systems have reshaped the rehabilitation of completely edentulous patients, helping clinicians overcome these challenges and enhance the efficiency of patient-centered care.^1^ Digital technologies have been integrated into the fabrication of both tissue- and implant-supported complete dentures, encompassing diagnostic procedures, implant-prosthetic planning, and the execution of definitive restorations.^2^ Accordingly, the adoption of new tools, such as intraoral scanning, facial scanning, and photogrammetry can streamline the acquisition of digital records^3^ and increase the predictability of prosthetic outcomes through CAD-guided design and CAM workflows, using either additive or subtractive processing techniques.^4^

Conventional approaches remain prone to errors, as they involve multiple stages for capturing impression pouring and potential dimensional changes of impression and dental cast. These steps often require several clinical visits, resulting in longer chair time and higher costs. To address these limitations, straightforward protocols and techniques have been developed within the fully or hybrid digital workflows to optimize full-arch prosthodontic rehabilitation, targeting specific clinical procedures, virtual design and acquisition of prosthetic components, or fabrication of definitive prosthesis.^5^,^6^,^7^ Nuytens et al.^6^ proposed a user-friendly scanning method for transferring and articulating a maxillary edentulous arch rehabilitated with multiple implants, employing a modified scan body system and facial scanning to incorporate all necessary steps for fabricating an implant-supported fixed complete dental prosthesis (ISFDP) prototype. Similarly, Altoman et al.^7^ described a digital workflow for zirconia ISFDP that eliminates the need for a provisional conversion prosthesis and conventional steps using a 3-appointment protocol with milled verification jigs to record jaw relations. Although digital workflows provide clear advantages for edentulous patients, including fewer appointments and simplified clinical and laboratory procedures, certain clinical situations still pose challenges in maximizing treatment efficiency and predictability.

Previous studies have evaluated several aspects of complete denture fabrication within digital workflows, including clinical performance and patient-reported outcomes,^8^ the accuracy of digital impressions compared to conventional impressions for complete dentures,^9^ methods for recording maxillomandibular relationship^10^ and techniques for definitive impressions of completely edentulous arches.^11^ However, the authors are unaware of a scoping review investigating the clinical implications of digital workflow in implant-supported and removable full-arch rehabilitations from the dental practitioner's perspective. Scientific evidence remains unclear regarding the accuracy, time- and cost-effectiveness, and clinician satisfaction of digital workflows for the rehabilitation of edentulous arches with both tissue- and implant-supported complete dentures. Understanding their applicability in clinical procedures is essential for minimizing operational errors, ensuring predictable outcomes, optimizing patient management, and supporting informed decision-making. Therefore, this scoping review aimed to map the available evidence on the clinical applicability and implications of digital workflow in various interventions for fixed and removable full-arch rehabilitation in completely edentulous patients, with a focus on workflow-related operational outcomes and clinician-reported measures.

## Methods

### Protocol and registration

This scoping review was reported in accordance with the recommendations of the Preferred Reporting Items for Systematic Reviews and Meta-Analyses extension for Scoping Reviews (PRISMA-ScR) criteria^12^ and was recorded on the Open Science Framework platform (https://osf.io/xw2gb).

### Research question and PCC strategy

The guiding question was "What is the current evidence on digital workflows for full-arch rehabilitations and their implications for clinical practice?". For this study, the PCC strategy was applied, in which (P) the population consisted of completely edentulous patients, (C) the context involved digital workflow applied to tissue- and implant-supported full-arch prostheses, and (C) the concepts included the clinical implications of workflow-related endpoint and the clinician's perspective.

### Eligibility criteria

The eligibility criteria for selecting studies were prospective or retrospective clinical studies and review articles addressing the clinical implications of workflow-related performance indicators and the dental practitioners' perceptions considering the digital workflow. Clinical implications were defined as clinician-centered outcomes (clinical satisfaction) and workflow efficiency-related operational aspects, including the number of visits, chairside time, feasibility, cost-effectiveness, and accuracy of clinical procedures or steps. Studies focusing exclusively on prosthetic outcomes, such as passivity, fit, occlusal records, complications, and survival rates without assessing clinical workflow or clinician-related parameters were excluded. Additional exclusion criteria comprised short communication, case reports, case series, book chapters, dental techniques or protocols, studies evaluating only conventional workflow or duplication techniques, patient management with terminal dentition, patient-reported outcomes, studies with an exclusively surgical focus, and without available full text.

### Information sources and search strategy

The electronic search was carried out by two independent reviewers (A.K.C.R., A.F.P.C.)) on five databases (PubMed/MEDLINE, Scopus, Web of Science, Cochrane Library, Embase). The search strategy combined controlled vocabulary terms (MeSH and Emtree) with free-text keywords. Articles published in Portuguese, Spanish, or English languages from January 2020 up to April 2026 were considered for inclusion in the study. Disagreements were discussed between the authors to establish a consensus. Supplementary manual searches were performed in the non-peer-reviewed sources (ProQuest - Dissertations and Theses and ClinicalTrials.gov) as well as in dental journals to identify published articles in the fields of Digital Dentistry and Prosthodontics. Furthermore, the reference lists of all included studies were screened to retrieve additional potentially relevant articles. The search strategy used in the databases is described in Table 1.

**Table 1:** Search strategy for databases.

**Database**	**Search strategy**
PubMed/MEDLINE	((((((complete denture[MeSH Terms]) OR (overdenture[MeSH Terms])) OR (implant-supported fixed prosthesis)) OR (jaw, edentulous[MeSH Terms])) OR (mouth, edentulous[MeSH Terms])) OR (complete edentulism)) AND (((((((cad cam[MeSH Terms]) OR (computer aided design[MeSH Terms])) OR (computer aided manufacturing[MeSH Terms])) OR (digital workflow)) OR (3d printing[MeSH Terms])) OR (milled)) OR (artificial intelligence[MeSH Terms])) Filters: from 2020 - 2026
Scopus	TITLE-ABS-KEY ("complete denture" OR "full denture" OR "overdenture" OR "implant-supported fixed dental prosthesis" OR "edentulous mouth" OR "complete edentulism" ) AND TITLE-ABS-KEY ( "digital workflow" OR "CAD-CAM" OR "computer-aided design" OR "computer-aided manufacturing" OR "additive technique" OR "milling technique" OR "3D-printed" OR "artificial intelligence" ) ) AND PUBYEAR > 2020
Web of Science	"complete denture" OR "full denture" OR "overdenture" OR "implant-supported fixed dental prosthesis" OR "edentulous mouth" OR "complete edentulism" (Topic) AND "intraoral scanner" OR "digital workflow" OR "CAD-CAM" OR "computer-aided design" OR "computer-aided manufacturing" OR "milling technique" OR "3D-printed" OR "artificial intelligence" (Topic) Publication Years: 2020-2026
Cochrane Library	MeSH descriptor: [Denture, Complete] OR [Denture, Overlay] OR [Dental Prosthesis, Implant-Supported] OR [Jaw, Edentulous] OR [Mouth, Edentulous] (explode all trees) AND MeSH descriptor: [Computer-Aided Design] OR [Printing, Three-Dimensional] OR [Artificial Intelligence] (explode all trees)
Embase	('complete denture'/exp OR 'implant supported prosthesis'/exp OR 'overlay denture'/exp OR 'edentulism'/exp OR 'edentulous jaw'/exp) AND ('computer aided design/computer aided manufacturing'/exp OR 'cad/cam software'/ exp OR 'three dimensional printing'/exp OR 'milling'/exp OR 'digital workflow'/exp OR 'intraoral scanner'/exp OR 'artificial intelligence'/exp) AND [2020-2026]/py

### Study selection and data collection

The articles were managed with an online review software (Rayyan; https://www.rayyan.ai) to facilitate the selection process, review of the articles, and data collection. After removing duplicates, two independent investigators (A.K.C.R., A.F.P.C.) screened the titles and abstracts based on the eligibility criteria. Studies identified as potentially relevant were subsequently underwent full-text assessment. The inter-examiner agreement was calculated using kappa scores.^13^ The data collection was conducted in two steps: a) descriptive summarization of the general characteristics of the each study, including author, year of publication, country, journal title, study purpose and design, clinical application and context, on a data extraction sheet, and b) main findings that explain how the studies were conducted, as well as the evaluated parameters, key outcomes, and clinical implications. The variables were extracted by an independent investigator (A.K.C.R.), while another (A.F.P.C.) was responsible for checking them.

### Data synthesis

The extracted data were organized and charted into structured tables using Microsoft Excel spreadsheets to facilitate data synthesis. Study characteristics, methodological aspects, evaluated outcomes, and key findings were descriptively summarized. A narrative synthesis approach was adopted to present and interpret the evidence according to the objectives of this scoping review.

## Results

### Study selection

**Figure 1:**
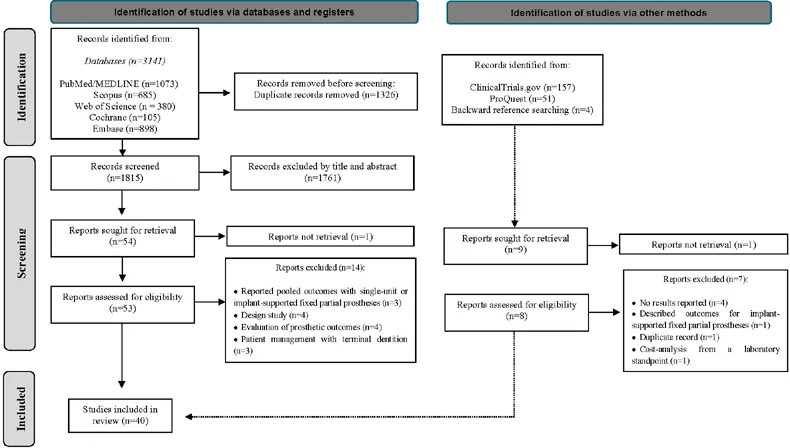
Flow diagram for the study selection process.

The electronic search retrieved 3141 records. After removing duplicates, 1815 articles remained. Of these, 53 full-text articles were screened according to the eligibility criteria. In total, 40 studies published between 2020 and 2026 were included (Figure 1). The kappa coefficient^13^ for the articles selected from PubMed/MEDLINE (0.92), Scopus (0.96), Web of Science (1.0), Cochrane Library (1.0), and Embase (1.0) evidenced "almost perfect agreement".

### Study characteristics

**Figure 2:**
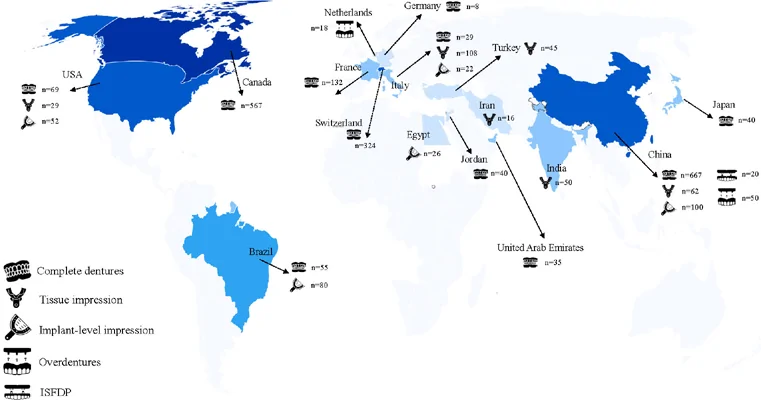
Worldwide geographical distribution of clinical applications using digital workflows. ISFDP, implant-supported fixed dental prosthesis.

Table 2 demonstrates the descriptive characteristics of included studies. In this review, the study designs included systematic reviews (n = 11),^14^,^15^,^16^,^17^,^18^,^19^,^20^,^21^,^22^,^23^,^24^ retrospective studies (n = 5),^25^,^26^,^27^,^28^,^29^ clinical trials (n = 8),^30^,^31^,^32^,^33^,^34^,^35^,^36^,^37^ prospective studies (n = 4),^38^,^39^,^40^,^41^ pilot (n = 4),^42^,^43^,^44^,^45^ diagnostic accuracy (n = 1),^46^ a cohort study (n = 2),^47^,^48^ cross-sectional (n = 1),^49^ and within-subjected comparative study (n = 4).^50^,^51^,^52^,^53^ A total of 3603 completely edentulous patients were evaluated, with sample sizes ranging from 8^32^ to 511^22^ for both digital and conventional workflows (Tables 2 and 3). Several clinical applications were identified, including complete dentures, tissue-and implant-level impression procedures, implant-supported fixed dental prosthesis (ISFDP), and overdentures (IODs) (Table 3). Figure 2 illustrates the geographic distribution of clinical applications associated with digital workflow across sixteen countries.

**Table 2:** Descriptive data for included studies.

Author (year)	Country	Journal	Objective	Study design	Number of patients	Clinical application	Clinical context
AI-Kaff & AI Hamad (2024)^30^	Jordan	Journal of Prosthodontics	To assess the clinical outcome and patient satisfaction of additively manufactured complete dentures with IOS and hybrid cast digitization in comparison with conventional complete dentures	Controlled clinical trial	20 bimaxillary edentulous patients	Complete dentures	Clinician's perception
Casucci et al (2025)^25^	Italy	Journal of Dentistry	To compare the clinical effectiveness and cost-efficiency of conventional and digital prosthodontic protocols, with a specific focus on milled and 3D-printed digital removable complete dentures	Retrospective study	60 edentulous patients (mean age 74.46 ± 8.03 years)	Complete dentures	Time-efficiency and costs
Casucci et al (2023)^14^	Italy	International Journal of Prosthodontics	To evaluate accuracy and working time of digital impressions vs conventional impressions taken for removable prostheses on edentulous arches	Systematic review	76 edentulous patients	Tissue impression	Accuracy and time-efficiency
Arakawa et al (2022)^26^	Switzerland	The Journal of Prosthetic Dentistry	To compare the patients' burden for CAD-CAM and conventional dentures fabrication procedures	Retrospective study	32 edentulous participants (age 35-85 years)	Complete dentures	Time-efficiency and costs
Chocano et al (2023)^15^	Brazil	The Journal of Prosthetic Dentistry	To identify, compare, and synthesize the outcomes of published clinical studies related to complete denture fabrication, with respect to the differences between CAD-CAM technology and conventional techniques	Systematic review	27 patients	Complete dentures	Time and cost
Chen et al (2022)^48^	China	BMC Oral Health	To compare the short-term clinical outcomes of a digitally prefabricated implant-supported full-arch provisional prosthesis with those of a conventionally fabricated provisional prosthesis	Retrospective cohort study	39 patients (ranging from 29 to 82 years)	Implant-supported full-arch prostheses	Time
Chochlidakis et al (2020)^38^	USA	Journal of Prosthodontics	To compare the accuracy of digital and conventional maxillary implant impressions for completely edentulous patients	Prospective clinical study	16 patients	Implant-level impression	Accuracy
Clark et al (2021)^49^	USA	Journal of Prosthodontics	To evaluate if there is a difference in number of visits and remake rate when comparing conventionally fabricated and digitally fabricated complete dentures by dental students in a predoctoral student dental clinic	Retrospective cross-sectional study	314 patients	Complete dentures	Time-efficiency
Deng et al (2022)^44^	China	Journal of Dentistry	To evaluate a novel 3D-printed custom tray for impressions of edentulous jaws, and to compare it with conventional impression trays	Pilot study	15 completely edentulous patients	Tissue impression	Accuracy
Deng et al (2021)^36^	China	Journal of Advanced Prosthodontics	To establish a digital complete denture system which can reduce the number of visits, does not require specific supporting tools, has a wide range of applications, and has a low cost for those regions where CAD-CAM fabrication fees are more expensive than that of conventional processing	Clinical trial	40 edentulous patients (average age was 73 years old)	Complete denture	Accuracy, time-efficiency, and clinician's perception
Deng et al (2023)^43^	China	Journal of the American Dental Association	To compare the treatment outcomes and time efficiency between FSD and conventional complete denture restorations and propose suggestions for clinical use.	Pilot study	10 edentulous patients	Complete dentures	Time-efficiency, costs, and dentist's satisfaction
Eldabe et al (2025)^47^	Egypt	The Journal of Prosthetic Dentistry	To evaluate the degree of trueness of an IOS and an intraoral photogrammetry scanner (IPS) for complete arch implant-supported prostheses.	Cohort study	13 edentulous arches (11 patients)	Implant-level impression	Trueness and time-efficiency
El Osta et al (2025)^16^	France	The Journal of Prosthetic Dentistry	To evaluate the time efficiency and cost of conventional, hybrid, and completely digital workflows throughout the entire process of removable complete dentures fabrication	Systematic review	132 patients	Complete dentures	Analyses of cost and time-efficiency
Fouda et al (2024)^17^	Canada	Journal of Prosthodontics	To compare digitally fabricated complete dentures to conventionally fabricated dentures using patient- and clinician-reported outcome measures	Systematic review	208 patients	Complete dentures	Clinician-reported outcomes and time-efficiency
Fu et al (2024)^45^	China	Clinical Oral Implant Research	To compare the accuracy of intraoral scan system with prefabricated aids and stereophotogrammetry compared with open tray implant impression for complete-arch implant-supported fixed dental prostheses	Pilot study	15 patients with 22 edentulous arches	Implant-level impression	Accuracy and time-efficiency
Hack et al (2020)^39^	USA	Journal of Prosthodontontic Research	To evaluate the feasibility and accuracy of computerized optical impression making of edentulous jaws in an *in vivo* setting.	Prospective clinical study	29 edentulous patients	Tissue impression	Feasibility and accuracy
Jafarpour et al (2024)^18^	Canada	Journal of Oral Rehabilitation	To compare CAD-CAM CDs with the traditional ones in terms of patient and clinician-reported outcomes, post-insertion adjustment visits and costs.	Systematic review and meta-analysis	1223 patients	Complete denture	Clinician perception and time-efficiency
Karaca & Akca (2024)^37^	Turkey	BMC Oral Health	To compare different conventional and digital impression methods used for tissue-supported maxillary complete dentures.	Clinical trial	15 maxillary edentulous patients	Tissue impression	Accuracy
Lo Russo et al (2025)^50^	Italy	The Journal of Prosthetic Dentistry	To measure the potential distortion of intraoral scans of edentulous mandibular arches made with a 2-step scanning strategy and to assess their differences with conventional impressions	Within-subject comparative study	20 edentulous patients (20 mandibular edentulous arches)	Tissue impression	Accuracy
Nagar et al (2024)^31^	India	Journal of Pharmacy and Bioallied Sciences	To compare the efficacy, accuracy, and patient outcomes between conventional impression methods and intraoral scanning for the fabrication of complete dentures	Randomized clinical study	100 patients	Tissue impression	Time-efficiency
Peroz et a 1 (2022)^32^	Germany	The Journal of Prosthetic Dentistry	To evaluate the impact of the digital versus conventional production of complete dentures on oral health-related quality of life measures	Randomized, controlled, blinded crossover trial	16 participants (66.4 ±8.31 Years)	Complete denture	Time-efficiency
Pozzi et al (2023)^40^	Italy	Clinical Oral Implants Research	To assess accuracy of intraoral optical scanning (IOS) and stereophotogrammetry (SPG), complete- arch digital implant impressions in vivo	Prospective comparative study	11 edentulous patients (11 complete arches)	Implant-level impression	Accuracy
Vieira et al (2025)^19^	Brazil	MDPI - Applied Sciences	To analyze clinical studies that evaluated intraoral scanning versus conventional impression to obtain rehabilitation of full-arch fixed prostheses and removable.	Systematic review and meta-analysis	75 patients	Implant-level impression	Time-efficiency
Ohara et al (2022)^33^	Japan	Journal of Prosthodontic Research	To evaluate patient satisfaction with conventional dentures and digital dentures fabricated using 3D printing. This study also compared the patients' quality of life, number of visits, time required for definitive denture fabrication, number of adjustment appointments, and time required for denture stabilization after denture delivery as secondary outcomes.	Randomized controlled crossover trial	20 edentulous patients	Complete denture	Time-efficiency
Otake et al (2024)^27^	Japan	The Journal of Prosthetic Dentistry	To evaluate general patient satisfaction with complete dentures fabricated through the custom disk method. In addition, a comparative cost-effectiveness analysis was conducted for the custom disk method and conventional removable complete dentures.	Retrospective study	44 edentulous participants	Complete denture	Cost-effectiveness
Papaspirydakos et al (2023)^28^	USA	Journal of Prosthodontics	To compare the 3D deviations between full arch digital scans and conventional implant impressions for edentulous maxilla and mandibles.	Retrospective study	27 patients (36 edentulous jaws)	Implant-level impression	Accuracy
Srinivasan et al (2025)^42^	Switzerland	Journal of Dentistry	To explore the feasibility and reliability of measuring the vertical dimension of occlusion/rest on 3D facial scans of edentulous patients	Pilot study	19 edentulous patients	Complete dentures - Measurements of vertical dimension	Feasibility
Srinivasan et al (2021)^34^	Switzerland	Journal of Dentistry	To evaluate and compare the differences between milled and 3D-printed complete removable dental prostheses	Double-blind, randomized, crossover, clinical trial	15 edentulous patients (66.7 ± 8.0 years)	Complete dentures	Cost and clinician's perception
Srinivasan et al (2021)^20^	Switzerland	Journal of Dentistry	To compare CAD-CAM and conventionally constructed removable complete dentures	Systematic review and meta-analysis	146 patients	Complete dentures	Cost and time
Tew et al (2025)^21^	China	The Journal of Prosthetic Dentistry	To compare the cost-effectiveness, the frequency of clinical and postdenture delivery visits, patient satisfaction, and OHRQoL between conventionally and digitally fabricated complete dentures.	Systematic review and meta-analysis	489 patients	Complete dentures	Cost-efficiency
Van de Winkel et al (2025)^35^	Netherlands	Clinical Implant Dentistry and Related Research	To estimate the decline in costs for digitally designed and CAD-CAM fabricated lODs compared to conventionally fabricated lODs at comparable general health related quality of life.	Randomized crossover study	36 fully edentulous patients (mean age 62.8 years)	IOD	Cost
Ribeiro et al (2024)^46^	Brazil	Journal of Advanced Prosthodontics	To assess the accuracy and time efficiency of a digital method to draw the denture foundation extension outline on preliminary casts compared with the conventional technique.	Diagnostic accuracy study	14 patients (28 preliminary edentulous casts)	Complete dentures - Outlining of denture foundation	Accuracy and time-efficiency
Zhang et al (2023)^51^	China	The Journal of Prosthetic Dentistry	To evaluate the accuracy (trueness) of photogrammetric imaging for complete arch implant-supported prostheses by comparing photogrammetric imaging with verified conventional splinted impressions.	Within-subject comparative study	14 completely edentulous jaws	Implant-level impression	Accuracy
Zhao et al (2025)^52^	China	Frontiers in Bioengineering and Biotechnology	To evaluate prosthodontists' satisfaction and efficiency of custom tray fabrication methods in completely edentulous mandibular jaw patients.	Within-subject comparative study	32 edentulous patients	Tissue impression	Clinician's satisfaction and time-efficiency
Yan et al (2023)^41^	China	The Journal of Prosthetic Dentistry	To compare the accuracy of IOS and SPG for complete arch implant scans with laboratory scanning as reference and to evaluate the passive fit of frameworks fabricated with SPG.	Prospective study	17 patients (21 arches and 4, 6 or 8 implants)	Implant-level impression	Accuracy
Smith et al (2021)^29^	USA	Journal of Prosthodontics	To evaluate the cost savings, if any, of fabricating complete dentures digitally versus traditionally through an outside lab	Retrospective study	30 patients	Complete dentures	Costs and time-efficiency
Thu et al (2024)^22^	China	The Journal of Prosthetic Dentistry	To evaluate the clinical and laboratory procedures for digital complete dentures, their outcomes, and associated complications	Systematic review	NR	Complete dentures and IOD	Clinician-reported outcomes, accuracy, and time-efficiency
Muehlemann et al (2026)^23^	Switzerland	Journal of Prosthodontics	To compare digitally designed and manufactured removable complete dentures with conventionally fabricated ones in terms of cost-efficiency.	Systematic review and meta-analysis	184 patients	Complete dentures	Cost-efficiency
Khiavi et al (2026)^53^	Iran	Health Science Reports	To compare the dimensional accuracy of digital intraoral scans and conventional definitive impressions for the fabrication of complete removable dentures in edentulous patients.	Within-subject comparative study	10 edentulous patients (n=16 arches) (65.4 ± 7.2 years)	Tissue impression	Accuracy
Sivaswamy et al (2026)^24^	United Arab Emirates	The Journal of Prosthetic Dentistry	To evaluate the clinical outcomes of 3D printed complete dentures against conventional and milled dentures.	Systematic review and meta-analysis	NR	Complete dentures	Clinician satisfaction

CAD-CAM, computer-aided design and computer-aided manufacturing; FSD, functional suitable digital; IOD, implant overdenture; IOS, intraoral scanner; IPS, intraoral photogrammetry scanner.

**Table 3:** Key findings regarding clinician-reported outcomes.

Author (year)	Groups	Number of procedures	Data collection methods	Prosthetic steps	Evaluated parameters	Key findings	Clinical implication
AI-Kaff & AI Hamad (2024)^30^	AMI: CDs manufactured with IOS + 3D-printed AMH: CDs manufactured with hybrid cast digitization + 3D-printed CCD: CDs manufactured with conventional impression and technique	CCDs (n=20) 3D-printed dentures (n=40)	Clinically assessed by a prosthodontist (>20 years of experience)	CCD: Preliminary and definitive impressions, adjustment of occlusal rims and MMR, try-in, and delivery. Trial dentures of the CC group were scanned for occlusion registrations of the AM 1 and AMH groups and were used to guide the designing process (ExoCAD). Both AMI and AMH dentures were 3D printing and post-processing.	Denture base contour, lip support, occlusion, extension, phonetics, prognosis, teeth arrangement, esthetic, centric relation, vertical dimension, stability, retention, and overall domains	The CCD group had higher quality in tooth arrangement criteria than AMI (p=0.04), and AMH (p=0.04), and higher quality than AMI for centric relation (p=0.014), esthetics (p=0.025), and mandibular CD retention (p=0.005). The overall scores for CCD (55.5) were significantly higher than AMI (52.8), whereas CCD was comparable to AM H (54.7).	The clinical outcomes for hybrid dentures are comparable, suggesting that additive manufacturing is feasible for clinical application. However, the AMI have generally a lower quality than the CCD for both arches and lower quality than hybrid dentures for the mandibular arch. Teeth arrangement of both additively manufactured dentures is clinically inferior to the conventional denture, and the retention of dentures with IOS are inferior to the CCD and AMH.
Casucci et al (2025)^25^	Conventional and digital complete dentures	CCDs (n=30), milled dentures (n=14), and 3D-printed dentures (n=15)	Laboratory costs, including material costs, technician time, and overheads) and operational time (follow-up time; chairside time)	CCD: preliminary and definitive impressions, MMR, try-in, and delivery (4 appointments). DDs: patient's existing denture was used as a reference. The border molding, if necessary, was registered. The definitive impressions and MMR were scanned. A digital model of the new dentures was created (Dental systems, 3Shape) and try-in were 3D-printed. The digital dentures were finalized through either milling or 3D printing.	Chairside time (treatment duration), follow-up time, and laboratory costs	***Chairside time:*** DDs: 154.31 ±13.19 min CCDs: 218.00 ±20.75 min (p<0.0001) ***Laboratory costs:*** DDs: €378.79 ±137.46 CCDs: €459.15 ±63.72 (p=0.005) No significant differences were found between the groups in follow-up time (p=0.587) or the number of follow-ups required (p=0.694), suggesting similar post-treatment follow-up.	Digital removable dentures offer a practical and efficient alternative to conventional dentures, particularly in terms of reducing chairside time and laboratory costs.
Casucci et al (2023)^14^	Comparison: impressions taken with conventional techniques Intervention: digital impression with IOS	Conventional impression (n=88) and digital impression (n=88)	Searches carried out in 4 databases -6 studies published from 2018 up to 2020	NA	Working time and accuracy	The highest discrepancies were observed in soft palate (357 ±89 urn - 860 ±77 urn) and peripheral seal zone (posterior: 447 ±89 μm; anterior: 659 ±99 μm). Mean value of 186 seconds was reported as scanning time; however, information on patient preparation for digital scanning was not mentioned.	The IOS resulted in a high level of accuracy in attached mucosa, but differences in the peripheral areas could be expected when compared with conventional impressions. It is not possible to draw any conclusions on scanning time because of limited information.
Arakawa et al (2022)^26^	Conventional and digital complete dentures	CCDs (n=16) and CAD-CAM dentures (n=16)	Financial records (dentist's fees and materials, dental laboratory costs, and total costs), and operational time tracking (production, post-delivery and recall)	NR	Treatment duration, costs, and need for post-delivery modifications	Laboratory costs of CAD-CAM were significantly lower than CCDs (p<0.001), but clinical fees were similar between groups (p=0.596), resulting in a reduction in the overall total costs for the CAD-CAM (p=0.011). No statistically significant difference regarding the treatment duration (p=0.889), number of clinical visits (p=0.945), and number of adjustments (p=0.675) were found.	The digital treatment was less expensive in terms of overall costs, laboratory costs, and fewer clinical visits, suggesting that they might replace conventional dentures, reducing clinical treatment time and costs. Both complete dentures demonstrated similar number of treatment adjustments.
Chocano et al (2023)^15^	Comparison: CDs by using digital and conventional methods Intervention: digitally manufactured CDs	CCDs (n=27) and digital dentures (n=27)	Searches carried out in 5 databases - 2 studies published from 2015 up to 2018	NA	Time and cost	The conventional technique required significantly more clinical time than digital manufacturing of CDs. The laboratory and overall cost of CAD-CAM denture protocol was significantly lower than conventional protocol, despite material costs for this protocol being higher.	The clinical time and overall cost of digital dentures were lower than those for conventional CDs.
Chen et al (2022)^48^	Digitally prefabricated and conventionally fabricated provisional ISFDP	Conventional ISFDP (n=19) and digital ISFDP (n=20)	Operational time	Digital group: provisional prosthesis was stabilized with pins. Minor modifications were made to ensure passive fit and the provisional prosthesis with a metal framework reinforced acrylic resin-based restoration was delivered. Conventional group: impression and MMR were taken, and a wax-up prosthesis was made. So, provisional full-arch acrylic resin dentures were manufactured and fixed to the implants.	Restorative time	Digital ISFDP: 116.16 ±16.61 min Conventional ISFDP: 242.11 ±30.14 min (p<0.05).	The digital prefabrication technique involving an implant-supported full-arch provisional prosthesis might be a viable treatment option in edentulous patients due to the simplified restorative procedures.
Chochlidakis et al (2020)^38^	Scans of the verified conventional casts and virtual casts from intraoral full-arch impressions	Conventional impression (n=16) and digital impression (n=16)	Superimposition and best-fit algorithm software (Geomagic Control X)	Conventional final casts were scanned (Dental Wings) and intraoral full-arch digital scans (True Definition) were also obtained with scan bodies.	Accuracy of implant impression	The overall 3D deviation was 162 ±77 μm. There was a positive correlation between increased implant number and 3D-deviations, but there was no statistically significant difference (p=0.191).	The 3D accuracy of full-arch digital implant scans lies within previously reported clinically acceptable threshold (200 μm). Full-arch digital scans and a complete digital workflow in the fabrication of maxillary fixed complete dentures may be clinically feasible.
Clark et al (2021)^49^	Conventional and digital dentures	CCDs (n=242) and digital dentures (n=39)	Electronic health record	CCDs: preliminary and definitive impressions, MMR, wax tooth try-in, and placement (5 steps). DDs: preliminary and definitive impressions, Wagner try-in, and placement (4 steps).	Number of appointments, the number of postoperative visits, and any need for remakes	***Number of appointments:*** 50% of CCDs required 6 or more visits, compared to only 5% of DDs (p< 0.0001). ***Post-operative visits:*** CCDs: average of 2-3 visits DDs: 1-2 visits (p=0.028). ***Number of dentures remake:*** there was no statistical difference (p=0.190).	In the predoctoral clinic, DDs required fewer patient appointments from start to finish, and fewer post-operative appointments than CCDs. Fewer visits may be an important consideration for patients, especially those with limited access to care.
Deng et al (2022)^44^	Diagnostic denture and conventional tray impressions; non-pressure tray and final denture impression	Conventional impression (n=30) and digital impression (n=30)	Superimposition and best-fit algorithm software (Geomagic Qualify 3D)	The primary impressions and MMR were performed. A closed-mouth custom tray was designed (diagnostic CD). The conventional custom tray was designed based on the diagnostic denture and 3D-printed. A 3D-printed diagnostic denture and two custom trays were used to make final impressions. Border molding and definitive impression were performed with the diagnostic denture and conventional custom tray.	Accuracy (maxillary impression - primary and secondary stress-bearing areas, relief area, and palatal vault; mandibular impression - primary and secondary stress-bearing areas)	Compared to the final denture impression (reference), diagnostic denture impression (RMS: 146 ± 24 μm) was closer to the reference than the conventional impression (RMS: 176 ± 47 μm), with a significant difference only in the secondary stress-bearing area. The border extension of the diagnostic denture impression was slightly longer than the conventional impression; however, the difference was not statistically significant.	The impressions made using the novel custom tray (diagnostic denture) were similar to those made with a definitive complete denture. However, no significant differences were observed when compared with conventional impressions. A digitally fabricated diagnostic denture can potentially be used to make final impressions in a clinical setting to reduce the number of visits and improve the efficiency of complete denture treatment.
Deng et al (2021)^36^	Digital complete denture (FSD) made by a prosthodontic chief physician and postgraduate student	CCDs (n=40) and digital dentures (n=40)	N-point registration for alignment (Geomagic Studio)	Primary impressions and provisional MMR, trial denture, definitive impression and MMR, 3D-printed dentures as a mold for manufacturing of CCDs.	Number of visits, return visit, accuracy, dentist's evaluation of complete dentures (tissue surface adaptation, retention, stability, and border extension)	***Number of visits:*** Before dentures insertion: - Chief physician: 3.1 times - Postgraduate student: 3.3 times Return visit: - Chief physician: 1.2 times - Postgraduate student: 1.6 times ***Accuracy:*** 3D comparison between the impression made via diagnostic dentures and the final dentures were 165 ±33 μm (upper jaw) and 139 ±31 μm (lower jaw). VAS ratings: chief physician (8.5- 9.6); postgraduate student (7.7- 9.5) (p>0.05).	FSD can simplify the complete denture restoration process and reduce two visits. The accuracy of impressions was within clinically acceptable range. FSD is also very friendly to novices, but there is still a certain degree of experience reliance.
Deng et al (2023)^43^	Digital (FSD) and conventional complete dentures	CCDs (n=10) and digital dentures (n=10)	Dentist's evaluation (retention, stability, occlusal stability, and margin extension)	CCDs: primary and definitive impressions, occlusal rim adjustment and MMR, conventional try-in denture using stock teeth and wax, and delivery. Digital method: primary jaw relations and esthetic information obtained in FSD. The diagnostic denture was used to obtain closed-mouth definitive impressions and MMR. 3D-printed complete denture as a mold for manufacturing of the complete denture by a conventional technique.	Clinician satisfaction, time-efficiency, costs	***Clinical operational time:*** DDs: 130.5 ±30.5 min CCDs: 158.5 ±29.6 min (p<0.05) ***Laboratory time:*** DDs:98.5 ±9.4 min CCDs:162.8 ±6.0 min (p<0.05) ***Return visits:*** DDs:1.4 ± 0.5 CCDs:1.7 ±0.5 (p=0.031) ***Dentist's satisfaction:*** DDs: 7.6 to 9.6 CCDs: 7.2 to 9.7 (p>0.05). The FSDs showed better occlusal stability (p=0.031). On average, the mandibular retention and stability of FSD were higher than CCDs, while the esthetics and maxilla retention were lower (p>0.05). ***Costs:*** DDs saved $9.50 (clinic) and $10 (laboratory).	FSDs have a comparable clinical effect to conventional dentures, can save time, is low cost and easy to operate, reducing 2 clinical visits.
Eldabe et al (2025)^47^	IOS and IPS impressions	Digital impression (n=26)	Superimposition and best-fit alignment algorithm (Mimics)	An unsplinted open-tray impression technique was made. A light-polymerizing tray material was used for splinting the impression copings extraorally on the primary cast. A custom impression tray was designed and 3D-printed. The sectioned open-tray impression copings were seated in the patient's mouth and splinted with a low-shrinkage flowable composite resin. The definitive cast was poured. Two digital scans were recorded (IOS and IPS).	Trueness (3D linear and angular deviations), and chairside scanning duration	***3D linear deviation:*** IPS: 30.7 ±18 μm IOS: 59.8 ±30 μm (p<0.001) ***Angular deviation:*** IPS: 0.78 ±0.27 degrees IOS: 1.4 ±0.44 degrees (p<.001) In the IOS group, angular deviation had a positive correlation with the type of arch (p=0.013). ***Chairside time:*** IPS: 14.1 ±1.5 min IOS: 18.2 ±3.6 min Conventional: 39 ± 3 min (p<0.001).	The IPS significantly improved the ease and accuracy of recording complete arch implant-supported prostheses. Unlike IOS, the trueness of IPS was not affected by the type of jaw.
El Osta et al (2025)^16^	Comparison: conventional protocol for the fabrication of CD Intervention: Digital and/or hybrid protocol for the fabrication of CD	CCDs (n=132) and digital dentures (n=132)	Searches carried out in 2 databases - 8 studies published from 2015 up to 2024	NA	Time and cost to fabricate complete dentures	Both digital and hybrid workflows reduced both clinical and laboratory time and overall costs compared with conventional workflows. The hybrid protocol required significantly less clinical time, (reductions ranging from 1 to 4 hours). DDs reduced laboratory time by 5 to 7 hours.	The findings suggest that digital and hybrid workflows for complete dentures fabrication were more time efficient, affordable, and cost effective than conventional methods.
Fouda et al (2024)^17^	Comparison: conventional CDs Intervention: digital CDs	CDs (n=106), milled dentures (n=50), and 3D-printed dentures (n=52)	Searches carried out in 3 databases -7 studies published from 2021 up to 2024	NA	Clinician-reported outcomes, clinical- and time-efficiency, denture quality	DDs had superior cost-effectiveness, time-saving and improved clinical efficiency. Although there were challenges regarding esthetics and occlusion, the quality of DDs was comparable and sometimes better than conventional dentures in specific areas.	A trend emerged that milled dentures performed better than printed dentures. Clinicians adopting digital technology into removable prosthodontics may have a learning curve to overcome, and they should consider the patient-clinician relationship in addition to clinical outcomes to achieve patient satisfaction.
Fu et al (2024)^45^	Conventional, IOS and SPG impression	Conventional impression (n=22) and digital impression (n=44)	Superimposition and best-fit algorithm of software (Geomagic Studio)	Conventional: splinted open-tray technique Multiunit abutment analogs attached to the copings and dental stones obtained. IOS: Scan bodies with prefabricated bar splints were screw-retained to the multiunit abutments. IOS captured the scan bodies and intraoral soft tissue simultaneously. SPG: stereophotogrammetric transfers screwed into multiunit abutments. The stereophotogrammetric camera captured the position of all the transfers. A second intraoral scan was made to capture the soft tissues.	Distance and angle deviations, chairside time	***Angle deviation (median):*** IOS: 0.40° SPG: 0.31° (p<0.01) ***RMS errors (median):*** IOS: 69 μm SPG: 45 μm (p<0.01) ***Chairside time:*** IOS: 14.71 ±2.86 min SPG: 10.49 ±3.50 min Conventional: 20.20 ±3.01 min (p<0.01)	The accuracy of SPG and IOS with prefabricated aids were both clinically comparable. IOS was the most efficient workflow, regarding chairside time, followed by SPG and conventional.
Hack et al (2020)^39^	Datasets of conventional impressions, stone casts, and computerized optical impressions	Conventional impression (n=56) and digital impression (n=29)	Superimposition and best-fit algorithm software (Geomagic Qualify 3D)	Conventional definitive impressions using custom tray Laboratory scanning of impressions and stone casts (D700) Computerized optical impressions using IOS (Lava Chairside or True Definition)	Feasibility and accuracy of impressions	**General (mean deviations):** Cast: 336.7 ±105.0 μm Conventional: 363.7 ±143.1 μm IOS: 272.1 ±168.5 μm **Color-coded superimposition:** Highest deviations (≤500 μm) in the areas of the soft palate, the sublingual areas, and the vestibule (peripheral seal zone).	The investigated scanners were not able currently to fully replace a conventional impression for the fabrication of a complete denture. Technical and software-related improvements are necessary to be able to capture mobile soft tissue sufficiently.
Jafarpour et al (2024)^18^	Comparison: Conventional CDs Intervention: CAD-CAM CDs	CCDs (n=805), milled dentures (n=208), and 3D-printed dentures (n=257)	Searches carried out in 4 databases - 10 studies published from 2012 up to 2022	NA	Overall clinician satisfaction, number of post-insertion adjustment visits, laboratory and total costs	**Clinician satisfaction:** Although slightly in favor of CAD-CAM, the pooled results showed no significant difference in the mean overall clinician satisfaction between the CAD-CAM and CCDs (p=0.07). **Number of post-insertion adjustment visits:** CAD-CAM CDs are comparable with CCDs (p=0.21). **Costs:** Laboratory costs for CAD-CAM CDs were 39.53% lower than the CCDs (p<0.001) and the total costs for DDs were 22.44% lower than CCDs (p=0.02).	CAD-CAM CDs are not inferior when compared to CCDs and can be suggested as a promising treatment modality for the edentulous patients, given the lower costs associated with treatment and the potential for better access to care for the elderly.
Karaca & Akca (2024)^37^	Conventional impressions: individual tray with ZOE and stock tray with irreversible hydrocolloid Digital impressions: IOS with Al-scan, IOS without Al-scan, modified technique	Conventional impression (n=30) and digital impression (n=4S)	Superimposition and best-fit algorithm software (Geomagic Design X)	Digital impression without and with Al-scan of edentulous jaw. Conventional preliminary impression and digitizing with IOS. Occlusal vertical dimension and MMR with modified technique (wax baseplate) determined. Complete edentulous jaw impression and MMR were scanned. Conventional preliminary impression and digitizing with IOS. Conventional definitive impressions were digitized with IOS.	Right and left vestibular areas, postdam area, palatal area, right and left matching area, entire surface, and borders	In the conventional groups, the mean deviation at the borders was statistically significant (p=0.01). Comparisons between 2 steps -conventional impression and digital impression without Al-scans exhibited no significant differences for all regions (p>0.05). In the digital group with and without Al-scans, the mean deviation in the palatal area was significant (p=0.03).	In maxillary edentulism, digital impressions have shown comparable results to conventional impressions, suggesting promising implications for clinical applications.
Lo Russo et al (2025)^50^	Conventional and digital impressions	Conventional impression (n=20) and digital impression (n=20)	Superimposition and best-fit algorithm of surface-matching software (Geomagic Wrap)	Digitizing of edentulous arches using IOS following the 2-step scanning strategy. Design and 3D-printed custom trays. Conventional definitive impression made and immediately scanned (TRIOS4).	6 interest regions of right to left retromolar pad, along the residual ridge crest	The mean 3D distance between the intraoral scan and conventional impression was statistically significant (p=0.003). No significant effect of the factor "side" was found for global 3D deviations between surface curve and new curve, which appeared to exclude distortion. Conversely, a significant effect was found for the factor "region" (p<.05).	Intraoral scans did not exhibit significant distortion in comparison with conventional impressions. Adequate management of the stability of tissues around the residual ridge, favoring accessibility for the scanner, can yield reliable intraoral scans of the edentulous mandibular arch.
Nagar et al (2024)^31^	Conventional and digital impressions	Conventional impression (n=50) and digital impression (n=50)	NR	Conventional impressions: Preliminary and definitive impressions Intraoral scanning: Intraoral impressions using an IOS	Total clinical time from impression to denture delivery	DDs: 14.2±1.8 days CCDs: 18.5±2.3 days (p<0.01).	The intraoral scanning process significantly reduced the total clinical time from impression to denture delivery.
Peroz et al (2022)^32^	Conventional and digital complete dentures	CCDs (n=8) and digital dentures (n=8)	Dentists and dental laboratory technicians estimated the clinical and technical procedures	Conventional method: preliminary and definitive impressions, MMR and occlusal wax rim adjustments, wax evaluation, and delivery. Digital method: Individualization of maxillary BD key, adjustment with BD plane definitive impression, individualization of mandibular BD key and definitive impression, delivery.	Working time to fabricate the DDs and CDs	***Working time:*** DDs: 4 hours CCDs: 10.5 hours Digital dentures needed only 2 visits, 1 hour less chair time, and 5 hours less time for the dental laboratory technicians.	Conventional dentures needed more time for clinical visits and technical fabrication than digital dentures.
Pozzi et al (2023)^40^	IOS, SPG, and conventional impressions	Conventional impression (n=ll) and digital impression (n=22)	Best- fit software algorithm (DTX StudioLab)	Conventional: master casts were poured from the conventional impression. Master casts were digitized (D2000) IOS: IOS impression was recorded with an intraoral scanner using implant scan bodies secured at the multiunit abutment level. SPG: SPG recording digital coordinates of scan bodies secured onto the multiunit abutments.	Accuracy (3D linear and angular deviations)	***3D linear deviation:*** IOS: 137.2 μm SPG: 87.6 μm (p=0.014) ***Angular deviations:*** IOS: 0.79° SPG: 0.38° (p<0.0001)	SPG experienced significantly higher linear and angular accuracy. No effect of type of arch or implant number was detected. SPG can be feasible for complete-arch digital impressions with caution, and rigid prototype try-in is recommended before screw-retained prosthesis manufacturing.
Vieira et al (2025)^19^	Comparison: Conventional impression Intervention: Intraoral scanning	Conventional impression (n=80) and digital impression (n=80)	Searches carried out in 4 databases - 2 studies published from 2016 up to 2019	NA	Procedure time for the rehabilitations	***Implant level:*** There was a significant difference between the groups, presenting a mean difference of -9.18 min (p<0.00001). ***Level of patients:*** There was a significant difference between the groups, presenting a mean difference of -9.02 min. ***Number of retakes:*** At the level of dental implants was statistically significant (RR: 3.19, p<0.02), as well as at the patient level (RR: 3.17, p=0.010).	There was a notable advantage of the digital method over the conventional approach at both scanned patient and implant levels (p<0.05). Nevertheless, the number of repeated procedures was lower with the traditional impression method (p<0.05). It is important to note that only a limited number of studies were included in this analysis.
Ohara et al (2022)^33^	Conventional and digital complete dentures	CCDs (n=20) and digital dentures (n=20)	A stopwatch was used to measure the patients' treatment time	CCDs: preliminary and definitive impressions, MMR, try-in, and delivery DDs: Definitive impression, determination of incisor length and gothic arch record, try-in, and delivery	Number of visits, time required for definitive denture fabrication, number of adjustment appointments	The number of clinic visits was significantly lower in patients with DDs. However, there were no significant differences in the number of adjustment sessions and the time required for denture fabrication between both complete dentures.	Although DDs involved fewer clinic visits, the time required for denture fabrication and for adjustments did not differ between the groups. Thus, DDs fabricated using 3D printing may have comparable practicality and efficacy to conventional ones.
Otake et al (2024)^27^	Conventional and digital complete dentures	CCDs (n=24) and milled dentures (n=20)	ICER (incremental cost per change in general patient satisfaction)	DDs: Digital scan and preliminary MMR, diagnostic denture, definitive impression and MMR, and delivery CCDs: preliminary and definitive impressions, MMR, wax denture evaluation, and delivery	Direct costs (labor, materials, and equipment expenses) and time	***Labor, material and equipment costs:*** different between the 2 methods (p<0.001). ***Total costs:*** DDs: 41104 JPY CCDs: 45276 JPY (p=0.004). ***ICER:*** -251.4. Time: 96.0 ±7.1 min for design, 833.2 ±44.5 min for printing and 870.4 ±93.1 min for milling.	Material and equipment costs were significantly higher, while labor and total costs were significantly lower for the digital dentures than for the conventional dentures. Considering ICER, the digital denture was more cost-effective than the conventional denture.
Papaspirydakos et al (2023)^28^	Conventional and digital impressions	Conventional impression (n=36) and digital impression (n=36)	Superimposition and best-fit algorithm of software (Geomagic)	Digital method: abutment level full-arch digital scans (TRIOS3) were obtained. Conventional method: connecting abutment level impression copings to the implant abutments. Splinted open-tray technique was employed and generated impressions were poured. Conventional stone casts were scanned (Dental Wings).	3D deviations	The cumulative 3D deviations between virtual casts from IOS and digitized stone casts from conventional implant impressions were found to be 88 ±24 μm. In the maxillary group, the average 3D deviation was 85 ±25 μm, compared to 92 ±23 μm for the mandibular group (p=0.444).	The 3D implant deviations found between the full-arch digital and conventional impressions lie within the clinically acceptable threshold (150 to 200 μm).
Srinivasan et al (2025)^42^	Traditional analog and 3D measurements of vertical dimension	Conventional method (n=19) and digital method (n=19)	Reliability analysis using intra-class correlation coefficient (ICC) and Bland- Altman analysis	Conventional: measurements obtained on the face with the jaws positioned at the rest position (without CDs, RVD) and at centric occlusion (with dentures, OVD), between the facial landmarks Digital: Participants' faces were scanned twice, using a face-scanner (prostheses in centric occlusion, and without the prostheses while maintaining the jaws in rest position).	Measurements of vertical distances (G - SP, PN - SP, and SN - SP)	Digital measurements were higher than analog measurements, and the mean paired difference ranged from -4.86±3.2 to 0.42±2.7 mm. All the differences were statistically significant, except for SN-SP (p=0.110).	Digital methods for registering the RVD in edentulous patients can introduce errors, resulting in occlusal errors and discomfort. Clinicians must, for the moment, still use conventional analog methods to ensure accurate RVD measurement for a successful denture therapy.
Srinivasan et al (2021)^34^	Digital complete dentures	Milled denture (n=15) and 3D-printed complete dentures (n=15)	Questionnaire (CDQE)	The clinical workflow was conventional (preliminary and definitive impressions, occlusal wax rim adjustments, MMR, clinical trial of teeth set up in wax, delivery) and comprised of 5-6 clinical visits. Only the processing technique was different.	Clinician's denture quality evaluation (CDQE) considering lip support, lower lip line, retention, stability, and balanced occlusion, and prosthodontic maintenance needs (time spent, clinical and laboratory costs).	There were no differences in the CDQE between the two groups. There were no maintenance costs for the milled dentures either during the planned or unscheduled recall visits. However, 3D-printed dentures required more maintenance visits (n=3), with the average required time of 5 and 10 mins. Moreover, 3D-printed dentures required more clinical time for adjustment (p=0.0003), and costs for the clinical honorarium than milled (p=0.021).	Both milled and 3D-printed dentures are valid treatment modalities for edentulous patients, with the latter performing interiorly regarding the time and costs involved with the prosthodontic aftercare.
Srinivasan et al (2021)^20^	Comparison: Conventional CDs Intervention: CAD-CAM CDs	CCDs (n=73) and digital dentures (n=146)	Searches carried out in 3 databases - 9 studies published from 2012 up to 2021	NA	Time-cost analysis	CAD-CAM required less chairside time (p=0.037) and CCDs were more cost-effective than the milled CDs for clinical costs (p<0.0001). However, milled CDs were more cost effective than CCDs for the overall and laboratory costs (p<0.0001).	CAD-CAM CDs demonstrated many advantages including fewer patient appointments and reduced clinical time manufacturing costs.
Tew et al (2025)^21^	Comparison: Conventional CDs Intervention: milled and 3D-printed CDs	CCDs (n=363) and digital dentures (n=156)	Searches carried out in 3 databases - 8 studies published from 2011 up to 2023	NA	Manufacturing costs and number of clinical visits	DDs require less clinical time with a lower total cost, despite higher material costs compared with the conventional fabrication technique.	Both milled and 3D printed CDs can be a cost-effective and efficient option for rehabilitating edentulous patients.
Van de Winkel et al (2025)^35^	Conventional and digital overdentures	C-IOD (n=18) and D-IOD (n=18)	Cost consequence analysis	Conventional: primary and definitive impressions, MMR, wax try-in denture, delivery Digital: Digital Smile Design using facial photography, IOS of soft tissue and upper/lower denture, test fit of the bar and trial overdenture, delivery	Costs made within healthcare and patient costs	***Costs made within healthcare (production):*** Total costs: D-IOD showed a reduction in initial total costs of 14.2% (€4700.33 vs. €4030.61; p<0,001), in treatment time of 41.1% (308.6 vs. 182.0 min; p<0.001), and in number of treatment sessions of 47.1% (5.68 vs. 3.0; p<0.001). ***Costs made within healthcare (maintenance and repair):*** C-IOD and D-IOD were similar for treatment time (125.91 vs. 102.27 min; p=0.103) as well as additional laboratory costs (€49.56 vs. €26.58; p = 0.551). **Costs on patient level** For each patient, €142.75 with a travel time of 719 min were needed. Together with a total treatment time of 308.64 min, and total repairtime of 125.91 min, this resulted in a production loss of a total of 1153.45 min (19.2 h) per patient.	Implementing a 3D workflow in the production of overdentures supplies patients with high-quality digital overdentures at lower costs.
Ribeiro et al (2024)^46^	Conventional and digital outlining of extensions of denture foundation on preliminary casts	Conventional method (n=28) and digital method (n=28)	Superimposition and best-fit algorithm of software (Geomagic Design X)	Conventional: Outlining of the entire basal seat of the denture was performed on preliminary casts and digitized. Digital: preliminary edentulous casts with no anatomical landmarks were digitized using a laboratory scanner (Shining 3D). The outlines were drawn using software (Dental Wings).	Accuracy of denture foundation extension outline on preliminary casts and time-efficiency	***Accuracy:*** ranged from 0.57 to 0.92. The buccal and labial frenulum showed a lower value in the maxilla (0.57); while the area between the retromolar pad and buccal frenulum (0.64) showed a lower score in the mandible. ***Working time:*** The time was significantly longer for the digital method (p<0.001).	The denture foundation extension outline exhibited sufficiently excellent accuracy for the digital method, except for the maxillary anterior region. However, the digital method required longer working time.
Zhang et al (2023)^51^	Photogrammetric imaging and conventional splinted impressions	Conventional impression (n=14) and digital impression (n=14)	Superimposition and best-fit algorithm of software (Geomagic Studio)	Photogrammetric imaging: Custom scan bodies (ICamBody) were connected to the multiunit abutments intraorally. Scan bodies were captured using photogrammetric camera (ICam4D) Conventional: Splinted open-tray impressions were made in custom trays, and the working casts were obtained.	Distance and angular deviations	The overall distance deviation of photogrammetric imaging was 70 ±57 μm, significantly lower than the clinically acceptable level of misfit (p<0.001). The overall angular deviation was 0.432 ±0.348 degrees. Jaw was not correlated with distance or angular deviations either (p=0.190; p=0.209, respectively).	The accuracy (trueness) of photogrammetric imaging was within a clinically acceptable range of errors (150 μm). Jaw (maxilla or mandible) had no significant effect on the accuracy of photogrammetric imaging.
Zhao et al (2025)^52^	Conventional impression compound trimming technique, CAD and 3D printing technique, light-cured resin technique	Conventional impression (n=64) and digital impression (n=32)	Questionnaire and manual fabrication times documented using digital chronometer	**Conventional impression compound trimming technique:** preliminary impression, border and tissue surface trimmed, border molding and definitive impression **Light-cured resin technique:** preliminary impression, cast coated with light-cured resin and trimmed, and border molding and definitive impression **CAD and 3D printed technique:** preliminary impression, design of border extensions, and 3D printing (polylactic acid)	Clinician's satisfaction and operational time	Dimensional accuracy and border adaptation showed differences among groups (p<.001). Both impression quality and procedural efficiency showed statistically significant performance improvements (p<.001). CAD and 3DP approach receiving the highest overall satisfaction scores, with statistically significant advantages over conventional methods. CAD and 3D printing method significantly reduced manual fabrication time (p<.001) and achieved the best stability and retention (p<.05).	The CAD and 3D printing technique enhances user satisfaction, precision, and efficiency, reducing labor intensity while maintaining or enhancing the quality of impressions. However, the learning curve for digital workflows can impact clinical adoption.
Yan et al (2023)^41^	Laboratory scanning (LS) of a gypsum cast from a conventional impression, IOS, and SPG	Conventional impression (n=21) and digital impression (n=42)	Superimposition and best-fit algorithm of software (Geomagic Control X)	**LS group:** splinted open-tray splinted impressions. The definitive casts were digitized with laboratory scanner (E4; 3Shape). **IOS group:** scan bodies were scanned with an intraoral scanner (CS3600; Carestream). **SPG group:** scan bodies (ICamBodies, lmetric4D) were hand tightened onto the abutments and stereophotogrammetric scanner registered the 3D locations of the implants. Then, scan bodies were replaced by registering the soft tissue contours with the intraoral scanner (CS3600; Carestream). The digital cast generated (ExoCAD) was used to design the titanium framework of the ISFDPs.	Accuracy of IOS and SPG	The 3D discrepancies between SPG and LS ranged from 2.70 μm to 92.80 μm, whereas IOS and LS ranged from 21.30 μm to 815.60 μm.	The SPG technique was more accurate than IOS and, therefore, may be an alternative to laboratory scanning for complete arch implant scans and fabrication of titanium frameworks with passive fit. The accuracy of IOS was slightly affected by implant number and did not reach the clinical acceptance threshold for complete arch implant scans (150 μm).
Smith et al (2021)^29^	Digital complete dentures in a dental school context (all milled, hybrid of 3D printing and milling final product, and traditional method)	CDs (n=30) and digital dentures (n=30)	Electronic health records	Four-step workflow based upon the Ivoclar Vivadent process, including impressions and definitive impressions, centric bite relationship, try-in visit, denture insertion visit. Conventional workflow: 5-visit fabrication process (preliminary and definitive impressions, MMR, wax try-in and denture insertion visit).	Chair time costs and overall savings	A significant cost savings was achieved, both in terms of material and chair time costs when compared to traditional laboratory fabricated complete dentures. Using 3D printing for the preliminary steps resulted in cost savings ($448.97 vs $298.33). All milled approach achieved a profitability of $121.48 per hour, while hybrid workflow achieved $143.00 per hour, representing increase of profitability of 76.06% and 107.4% compared with traditional method, respectively. Student and faculty satisfaction with the digital denture process has been consistently high.	In this cost-benefit analysis at University setting, the digital denture protocol as compared to the CCD protocol was shown to be more cost-effective, yielding as much as a 65% savings over the traditional outside lab cost.
Thu et al (2024)^22^	Comparison: Conventional denture workflow Intervention: Digital CDs	Digital CDs (n=461) and digital lODs (n=50)	Searches carried out in 3 databases - 27 studies published from 2012 up to 2022	NA	Accuracy (denture base, definitive CDs or lODs, trial dentures, digital scans of impressions of edentulous jaws), clinician-reported outcomes and time-efficiency (clinical visits, recall visits, laboratory and clinical time)	***Accuracy:*** - Maxillary denture base: maximum discrepancy to the tissue surfaces ranged from 600 μm to 700 μm, considering the thickness of the fit-checking material. - Denture base and stone casts or IOS or digital images of the dentures: mean discrepancy were all below 500 μm. - The mean discrepancy between IOS and conventional impression mean discrepancy was less than 200 μm, while that to stone cast was less than 900 μm. ***Clinician-reported outcomes:*** - Higher ratings for retention and stability for milled maxillary CDs compared with conventional ones. - Stability of digital CDs was satisfactory. Retention was observed in 96.7% of milled and 86.7% of 3D-printed CDs. - Overall satisfaction (100mm-VAS): baseline (91.5) and 1-year follow-up (79.3) for milled CDs. ***Time-efficiency:*** - Fewer clinical visits for the fabrication of digital CDs (preliminary impression and trial placement may be skipped, resulting in 2 to 4 fewer visits). - Reduction in clinical time (58 to 233 minutes) when compared with conventional workflow; laboratory time was reduced up to 5 hours. - Mean recall visits for postoperative review or denture adjustment were 1 to 4.	Border-molded impression making for recording functional denture borders is more accurate when compared with IOS. Clinician scores for retention and stability were better for milled CDs than for conventional ones. Clinical steps can be combined to save clinical time and laboratory worktime. Extra visits may be needed because patients or clinicians may be dissatisfied with the definitive digital CDs.
Muehlemann et al (2026)^23^	Comparison: Conventionally designed and manufactured CDs Intervention: Digitally designed and manufactured CDs	CDs (n=118) and digital dentures (n=113)	Searches carried out in 4 databases - 5 studies published from 2019 up to 2025	NA	Direct costs (laboratory, clinical, and total costs), time-or performance-related measures, including the number of treatment sessions.	Laboratory costs (MD: -239.7 USD; p=0.10) Clinical costs (MD: 74.4 USD; p=0.45) Total costs (MD: -357.7 USD; p=0.25) Treatment sessions (M D: -1.5; p=0.35). Operator experience influenced clinical costs (p<0.0001) and the number of sessions (p=0.0001). Chairside time: Less experienced operators tend to require more chairside time and adjustments, contributing to increased laboratory and clinical costs.	Digital and conventional workflows for CDs fabrication demonstrated comparable cost-efficiency. Digital workflows may reduce the number of sessions; however, workflow selection should be guided by clinical feasibility, clinician and dental technician experience, and resource availability rather than assumptions of superior cost-efficiency.
Khiavi et al (2026)^53^	Direct intraoral scan (IOS) and scan of conventional definitive impressions (3D-printed custom tray - CI3D; acrylic special tray-CI).	Conventional impression (n=32) and digital impression (n=16)	STL files reversed (Geomagic Design X) and aligned using best-fit algorithms (Geomagic Control X)	**Group IOS:** An optical scan was made using an IOS (CS3800). **Group CI3D:** Using the generated STL files from IOS, special trays were designed in the Exocad software and fabricated using a resin material. Custom tray functionalization or border molding and definitive impressions were performed. The impressions were digitally scanned (CS 3800). **Group CI:** Initial impressions were made and poured. Custom trays were fabricated, functionalization was executed, and definitive impressions were made. The definitive impressions were digitally scanned.	Dimensional discrepancies (absolute 3D distance and RMS deviation)	No statistically significant differences were found between CI3D and CI in their deviations from the IOS reference. **Dimensional accuracy:** *Mean deviation:* - Maxillary: -0.11 ± 2.63 mm (IOS-CI); -0.55 ± 1.44 mm (IOS-CI3D) (p=0.55) - Mandibular: 0.10 ± 1.56 mm (IOS-CI); 0.32 ± 1.72 mm (IOS-CI3D) (p=0.60) *RMS deviation:* - Maxillary: - 2.44 ± 1.02 mm (IOS-CI) and 2.43 ± 1.06 mm (IOS-CI3D) (p=0.96) - Mandibular: 2.51 ± 0.63 mm (IOS-CI); 2.32 ± 0.62 mm (IOS-CI3D) (p=0.52)	Within the limitations of this clinical study, which included edentulous ridges with minimal to moderate bone loss (Cawood and Howell Class III), both conventional definitive impressions demonstrated comparable dimensional accuracy to IOS.
Sivaswamy et al (2026)^24^	Comparison: Conventional and milled CDs Intervention: 3D printed CDs	CDs (n=3S) and digital dentures (n=3S)	Searches carried out in 4 databases - 3 studies published from 2012 up to 2024	NA	Clinician satisfaction score	Both 3D printed and CCD groups showed no difference (p=0.43).	The clinician satisfaction was similar between 3D printed and CCDs. The methods could be routinely used in clinical practice, with the choice primarily guided by economic factors and individual patient preference.

Al, artificial intelligence; AMH, additively manufactured with cast digitization; AMI, additively manufactured with intraoral scanning; BD, Baltic system denture; C-IOD: conventional overdenture; CAD-CAM, computer-aided design and computer-aided manufacturing; CCDs: conventional complete dentures; CDs, complete dentures; CDQE, clinician's denture quality evaluation; D-IOD, digital overdenture; DDs, digital dentures; FSD, functional suitable digital; G, glabella; ICER, incremental cost-effectiveness ratio; ISFDP, implant-supported fixed dental prosthesis; IOD, implant overdenture; IOS, intraoral scanning; IPS, intraoral photogrammetry system; MD: mean difference; MMR, maxillomandibular relationship record; OVD, occlusion vertical dimension; PN, pronasale; RMS, Root mean square; RVD, rest vertical dimension; SN, subnasale; SP, soft pogonion; SPG, stereophotogrammetry system; VAS, visual analog scale; ZOE, zinc oxide eugenol impression paste.

**Figure 3:**
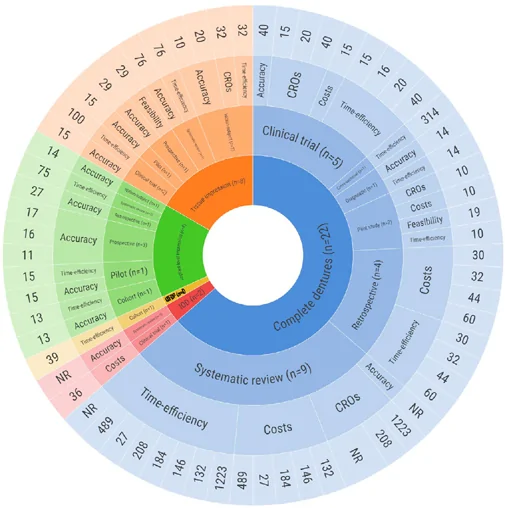
Sunburst chart. Segment size corresponds to the number of studies included. Inner ring represents clinical application, second ring represents study design, third ring represents clinical outcomes, and outer ring represents edentulous patients included (n). CROs: Clinician-reported outcomes. ISFDP: Implant-supported fixed dental prosthesis. IOD: Overdenture.

**Figure 4:**
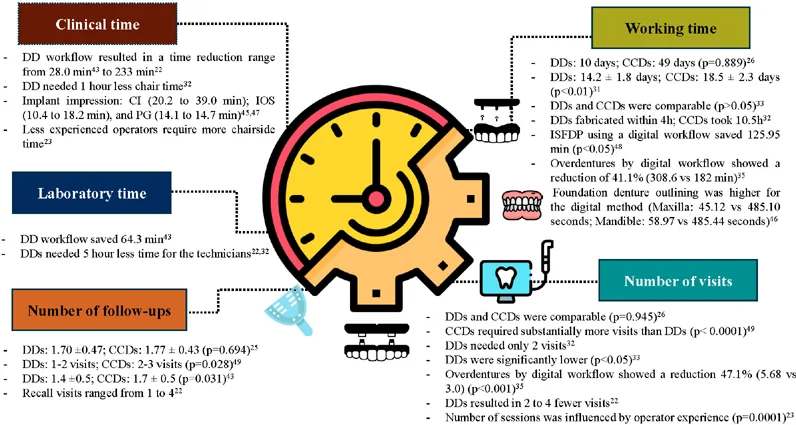
Time-efficiency of digital workflow compared to conventional method for solely clinical studies. CCDs, conventional complete dentures; CI, conventional impression; DDs, digital dentures; IOS, intraoral scanning; ISFDP, implant-supported fixed dental prosthesis; PG, photogrammetry system.

**Figure 5:**
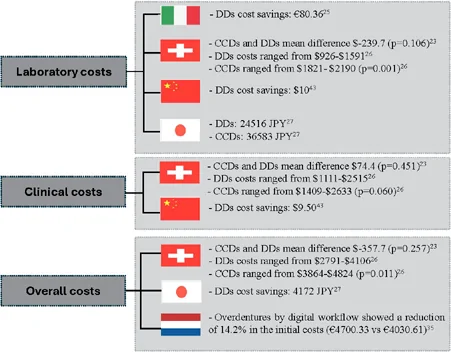
Cost analysis by country based on included clinical studies. CCDs, conventional complete dentures; DDs, digital dentures.

Most of the studies reported outcomes related to cost analysis,^15^,^16^,^18^,^20^,^21^,^23^,^25-27^,^29^,^34^,^35^,^43^,^48^ time-efficiency,^14-23^,^25-27^,^29^,^31-36^,^43^,^45-49^,^52^ and accuracy^14^,^22^,^28^,^36-41^,^44-47^,^50^,^51^,^53^ of clinical procedures or dental prosthesis fabrication process. Only two studies^39^,^42^ investigated feasibility outcomes. Figure 3 presents the distribution of studies according to clinical application, evaluated outcomes, and the number of edentulous patients included. Figures 4 and 5 demonstrate the time-efficiency and cost analysis, respectively, of digital workflow compared to conventional ones based on included clinical studies.

### Workflow-related operational parameters

#### Tissue-supported full-arch restoration

The accuracy of tissue impressions in edentulous arches was evaluated in eight studies.^14^,^22^,^36^,^37^,^39^,^44^,^50^,^53^ The highest discrepancies (≥ 500 μm) were reported in the peripheral areas, including the soft palate and the anterior border seal.^14^,^39^ Lo Russo et al.^50^ proposed a two-step scanning strategy for the edentulous mandible, yielding results comparable to conventional impressions with no detectable distortion. Likewise, digital impressions acquired using a single-step intraoral scanning (IOS) protocol without artificial intelligence-assisted scanning have demonstrated accuracy similar to that of a two-step conventional impression technique, supporting their potential applicability in clinical practice.^37^ Diagnostic denture impressions have also shown accuracy comparable to conventional methods;^36^,^44^ however, increased deviations were observed in secondary stress-bearing areas, likely due to their greater susceptibility to tissue displacement under functional pressure.^44^ Only one study^42^ assessed the reliability of vertical distance measurements in patients rehabilitated with complete dentures, demonstrating that digital measurements may introduce deviations leading to occlusal discrepancies, thereby recommending the use of conventional approaches for this clinical step. In contrast, another study^46^ investigated the accuracy of foundation denture extensions outlining and reported high accuracy in designing border extensions on preliminary casts (between 0.71 and 0.92), except for buccal and labial frenulum (maxilla) (0.57) and retromolar pad and buccal frenulum (mandible) (0.64).

A total of 22 studies assessed working time^14^,^15^,^16^,^17^,^20^,^21^,^22^,^23^,^25^,^26^,^27^,^29^,^31^,^32^,^33^,^34^,^43^,^46^,^52^ or the number of appointments.^18^,^22^,^23^,^25^,^26^,^32^,^33^,^34^,^36^,^43^,^49^ In general, digital workflows were significantly associated with substantial reductions in chairside time compared to traditional methods across different clinical applications.^15^,^16^,^17^,^20^,^21^,^22^,^25^,^31^,^32^,^43^,^52^ However, no significant differences were found when milled and 3D-printed complete dentures processing techniques were compared (p = 0.608).^25^ Additionally, three studies^18^,^26^,^33^ reported comparable overall treatment duration and/or number of clinical visits between the digital and conventional workflows. For dental prosthetic procedures, digital complete dentures reduced clinical time by up to 75.10% compared with traditional approaches.^22^,^25^,^43^ According to Srinivasan et al,^34^ both milled and 3D-printed dentures are suitable treatment modalities; however, 3D-printed dentures require more chairside time for adjustments. Ribeiro et al.^46^ conducted a diagnostic accuracy study evaluating denture base extension outlining by a prosthodontic expert and reported that the digital method was more time-consuming than the conventional approach.

Overall costs, including laboratory and/or clinical costs, were reported in 12 studies.^15^,^16^,^18^,^20^,^21^,^23^,^25^,^26^,^27^,^29^,^34^,^43^ Several studies^15^,^16^,^18^,^21^,^25^,^26^,^29^,^43^ reported cost reductions associated with the implementation of digital workflows, and one study^23^ stated that operator experience may influence clinical costs. Notably, the adoption of digital fabrication processes resulted in total laboratory cost savings ranging from approximately 9.21%^27^ to 39.49%^43^ compared with conventional workflows.^18^,^25^,^27^,^43^ However, one study found no significant differences in clinical fees between digital and conventional methods,^26^ whereas another demonstrated that milled dentures were more cost-effective in terms of overall and laboratory costs, while conventional dentures remained more cost-effective for clinical costs.^20^ Moreover, one study^34^ reported significantly higher clinical honorarium costs for 3D printed dentures compared with milled dentures (p = 0.021). In a dental school setting, Smith et al.^29^ demonstrated cost savings and higher hourly profitability with digital complete dentures. Specifically, fully milled and hybrid workflows combining 3D printing and milling achieved profitability of 76.06% and 107.4%, respectively, when compared with traditional methods.

#### Implant-supported full-arch restoration

Seven studies provided information related to the accuracy of implant impressions.^28^,^38^,^40^,^41^,^45^,^47^,^51^ Five studies used polyether-etherketone (PEEK) impression scan bodies,^28^,^38^,^40^,^41^,^47^ four studies incorporated ICam photogrammetry scan bodies to register the 3D location of implants (flag-type^40^,^45^ or flat-surfaced^41^,^51^), and one study^47^ utilized scan codes (scan flags) with a special dot orientation. Although discrepancies between digitized casts generated from conventional impressions and virtual casts from intraoral impressions have been demonstrated, the values for the 3D deviations of full-arch digital scans lie within clinically acceptable thresholds (up to 200 μm).^28^,^38^,^40^,^41^,^45^ Only one study^51^ reported that overall distance and angular deviations of stereophotogrammetry impressions (SPG) were lower than conventional splinted impressions. No significant correlations were found between the number of implants^38^,^40^ or jaw types^28^,^40^,^47^,^51^ and 3D deviations. Conversely, Eldabe et al.^47^ stated a statistically significant correlation between angular deviation and the mandibular arch when using IOS (p = 0.013). Increased errors or angular deviations were observed with IOS or laboratory scanning compared with SPG.^40^,^41^,^45^ However, the mean angular deviation obtained with IOS exceeded the theoretical limit of acceptable fit (1-degree) in only 1 study.^47^

All six studies reporting chairside time demonstrated a reduction when digital workflows were implemented. Implant-supported fixed dental prostheses and overdentures showed mean reductions in restorative time of 52.02%^48^ and 41.02%, respectively.^35^ Similarly, for implant-level impression procedures, digital techniques achieved time saving ranging from 27.17%^45^ to 63.84%^47^ with photogrammetry systems and from 48.06%^45^ to 53.33%^47^ with IOS, compared with conventional impression methods. Additionally, Van de Winkel et al.^35^ estimated the cost impact of CAD-CAM fabricated IODs and reported a 14.2% reduction in initial production costs compared with conventional IODs, resulting in significant decreases in treatment time (41.1%) and number of clinical sessions (47.1%) (p<0.001).

### Clinician-reported outcomes

#### Tissue-supported full-arch restoration

Clinician's perceptions were evaluated in nine studies that approached procedural efficiency for parameters such as tissue surface adaptation,^17^,^36^ retention,^17^,^22^,^30^,^34^,^36^,^43^ border extension,^30^,^36^,^43^,^52^ stability,^17^,^22^,^30^,^34^,^36^,^43^ esthetic,^17^,^30^ vertical dimension,^30^ balanced occlusion,^17^,^34^,^43^ and overall satisfaction.^18^,^22^,^24^,^52^ The findings varied considerably among studies. Two studies^17^,^30^ reported that overall satisfaction scores with digital workflow were significantly lower than conventional ones, mainly due to challenges related to esthetic and jaw registration. Conversely, both digitally fabricated complete dentures and impression procedures have demonstrated quality comparable to or better than traditional approaches.^17^,^18^,^24^,^52^ Prosthodontic chief physician and postgraduate student stated satisfied outcomes for Functional Suitable Digital (FSD) to accomplish complete denture restoration, demonstrating scores range from 7.7 ±1.0 to 9.6 ±0.7.^36^ Srinivasan et al.^34^ demonstrated that both milled and 3D-printed complete dentures were significantly better than existing conventional dentures. Although no statistical differences were found between digital dentures, the clinician's quality assessment attested the highest scores to the new dentures, which could explain the observed improvements in patient outcomes.

## Discussion

Digital workflows have become a widely adopted approach for the treatment of patients with complete edentulism. This concept encompasses the clinical application of virtual tools for diagnosis, treatment planning, and restoration, contributing to increased predictability of oral rehabilitation outcomes.^2^ In this context, the integration of healthcare technologies and network-based communication within CAD-CAM systems, together with artificial intelligence-driven solutions have enhanced clinical accuracy, streamlined clinical and laboratory processes, improved operational efficiency, and patient-centered care, while also promoting interdisciplinary collaboration among dental professionals.^5^ This scoping review mapped the clinical implications of digital workflow on interventions for fixed and removable full-arch edentulous rehabilitations, focusing on clinician-reported outcomes and workflow-related operational parameters.

Although splinted open-tray impressions remain the conventional standard for full-arch implant-supported dental prostheses,^45^ most studies have indicated that digital implant-level impressions for fully edentulous jaws may be clinically feasible. Specifically, intraoral photogrammetry impressions have demonstrated greater dimensional reliability in capturing landmark anatomical structures and implant positions, as evidenced by lower 3D linear and angular deviations.^40^,^41^,^45^,^51^ These findings suggest that SPG is more accurate than intraoral scanning impressions performed with vertical scan bodies and, therefore, could serve as a viable alternative for fabricating frameworks with passive fit. Although deviations reported in the included studies are within the clinically acceptable range of 150 - 200 μm, the clinical relevance of the differences between SPG and IOS should be interpreted considering the number and distribution of implants.

The clinical use of IOS for removable complete dentures remains challenging, particularly in accurately capturing movable mucosa, peripheral areas, and smooth surfaces of the edentulous arches. The lack of anatomical landmarks struggles with the image stitching process required to generate 3D datasets. Accurate reconstruction of files relies on overlapping regions; however, areas with poorly defined anatomical features, such as edentulous ridges or palatal surfaces, are prone to cumulative registration errors throughout the 3D model. Although IOS operates in a mucostatic manner without applying compressive forces, eliminating the shrinkage risks of conventional impression materials, strategies that carefully manage residual ridge tissues, combined with a two-step scanning approach for the edentulous mandible, may enhance the reliability of digital impressions and reduce distortions.^50^ Given the limitations of IOS in capturing the complex morphology of completely edentulous arches, further improvements in both hardware and software are essential to achieve accurate 3D datasets and minimize errors in digital impression procedures.

Overall, the majority of studies reported that digital workflows lead to significant reductions in clinical and laboratory time compared to traditional approaches. Digital technologies provide significant advantages in terms of operational sustainability and have demonstrated greater clinical efficiency in the prosthodontic field compared to conventional methods. The digital workflow simplifies restorative procedures while streamlining laboratory processes, allowing the fast design and manufacturing of prosthetic frameworks and complete dentures with high-quality outcomes. Moreover, the predictability and capacity to optimize scans in real time minimize the need for return visits and adjustments, further improving overall time-efficiency. Several factors hinder the widespread adoption of digital technologies, with a steep learning curve required for clinicians posing a major challenge. Consistent training and hands-on experience are essential for fully harnessing the precision, efficiency, and reliability offered by these systems.^46^ Despite their clinical benefits, successful implementation depends on proper education, training, and institutional support.

The incorporation of digital workflows into oral rehabilitation has notable implications for both clinical and laboratory processes, leading to lower operational expenses and improved time-efficiency for dental practices and prosthetic care for edentulous patients. The fabrication of digital dentures is associated with considerably lower overall laboratory costs due to the automation of multiple workflow steps in acquisition. Nevertheless, the initial investment in digital equipment and tools can be higher than that for conventional techniques, representing an upfront expense for the dental practice context.^27^ Cost-effectiveness and economic benefits should be analyzed with caution, as their feasibility may differ across countries depending on economic resources, healthcare infrastructures, and reimbursement policies. Time-related outcomes represent a more universal indicator of procedural efficiency, whereas monetary are inherently dependent on local healthcare economics (region-specific). Thus, clinicians and dental laboratories must carefully consider local financial conditions and cost-benefit ratios before implementing digital prosthodontic technologies on a broad scale.

Several studies have investigated the clinical-reported outcomes of digitally fabricated dental prostheses; however, the findings remain heterogeneous, and no conclusions can be drawn. Understanding conventional complete denture workflows is essential for clinicians before the complete transition to digital systems. Beyond technical proficiency, clinicians must also recognize the psychological and functional needs of each patient, assess limitations related to oral anatomy, cognitive state, and prior dental prosthesis experience, and clarify patient expectations. Emotional awareness of the impact of tooth loss and the challenges associated with edentulism is crucial for guiding treatment decisions and improving satisfaction. In this context, digital denture workflows underscore the crucial role of clinician-patient-technician interaction, addressing the patient's cognitive and emotional context before undertaking clinical prosthodontic care, which remains a guiding principle, ensuring that prosthetic interventions are both technically sound and aligned with the patient's purposes and perceptions.

### Practice-oriented clinical perspectives

Based on the evidence mapped in this scoping review and the authors' expertise, digital workflows appear to be more predictably integrated into clinical practice for implant-supported full-arch rehabilitations, particularly in implant-level impressions or CAD-CAM fabrication of fixed dental prostheses. These approaches may improve workflow standardization, laboratory communication, and manufacturing accuracy while reducing chairside and laboratory time. In removable prosthodontics, digital technologies have also demonstrated promising applications, especially for denture design, CAD-CAM manufacturing, and digital record storage. However, conventional or hybrid workflows may still represent more reliable approaches in specific clinical situations, such as border molding procedures, recording movable soft tissues, and managing complex maxillomandibular relationships, where current digital systems may present limitations in capturing dynamic anatomical details. Therefore, the integration of digital workflows into daily prosthodontic practice should be customized according to the clinical context, operator experience, patient-related factors, and the specific prosthetic requirements of each case.

### Trends for practice

Recent advancements in the integration of digital technologies have emerged as key trends in prosthodontics, enhancing treatment planning, clinical efficiency, and patient-centered outcomes. Although this review focused on clinician-centered operational outcomes, patient-reported outcome measures (PROMs) are increasingly recognized as important indicators of treatment success in oral rehabilitation. The clinical performance of dental prostheses is influenced by both prosthesis-related factors and subjective aspects associated with patient preferences and experiences. Clinician-reported outcomes are often related with technical skills and workflow execution, and operational efficiency, whereas patients tend to prioritize comfort, stability, esthetics, and masticatory satisfaction.^54^,^55^ Therefore, achieving a balance between clinician expectations and patient-centered outcomes is essential to optimize treatment outcomes in removable and implant-supported full-arch rehabilitations.

Tele-dentistry is increasingly being used for remote consultations and digital monitoring, expanding access to care and reducing treatment times, especially in rural and underserved areas, where dental professionals or access to oral healthcare are scarce.^5^ Augmented reality, combined with virtual occlusion planning and artificial intelligence, will allow clinicians to simulate, analyze, and optimize prosthetic designs with spatial and functional accuracy and predictability, thereby enabling highly customized treatment solutions. Moreover, the development of 3D-printed materials with improved mechanical strength, surface roughness, color stability, and biocompatibility will provide dental prostheses with better esthetic quality and higher longevity. Overall, these innovations represent a paradigm shift toward a fully digital workflow, reflecting a growing trend in contemporary prosthodontics toward efficiency, precision, and accessibility.

### Limitations and strengths

A potential limitation of the study is the possibility of double counting, as both primary clinical studies and systematic reviews were included, which may have led to overlap of underlying data. Therefore, the reported estimates of time and cost savings should be interpreted as reflecting overall trends, consistent with the exploratory nature of scoping reviews aimed at mapping the breadth of available evidence rather than providing pooled quantitative estimates. Additionally, the restriction to three languages may have introduced linguistic bias and limited the generalizability of the findings across different cultural contexts. This decision was made to maintain a controlled and standardized multilingual evaluation framework. The restricted inclusion period was adopted to capture the most current evidence in this rapidly evolving field. However, future studies should include a broader range of languages and cultural contexts to improve the robustness and applicability of the findings. Although this study focused on clinical evidence, the investigations included were heterogeneous in terms of methodological design, which limited direct comparisons between findings. Sample sizes also varied widely, with both small- and large-scale studies being analyzed together, potentially affecting the robustness and applicability of the results. Moreover, there was a lack of standardization in the instruments used to evaluate clinical satisfaction and procedural performance. Differences in scanning protocols, scanner models, digital techniques involving specific devices, and economic and geographic contexts across countries, including variations in monetary systems, are additional factors that should be considered when interpreting the results. Furthermore, the clinicians' learning curve and the operational context may influence the applicability and reproducibility of the findings.

## Conclusions

Based on the findings of this scoping review, the following conclusions were drawn:

a. The digital approach provides accurate results for full-arch implant-level impressions, with higher accuracy demonstrated by photogrammetry compared to IOS or conventional impressions; however, capturing tissues in edentulous arches remains challenging, particularly in areas with movable mucosa and poorly defined anatomical landmarks.
b. Digital workflow offers time- and cost-efficiency for fixed and removable full-arch rehabilitation of edentulous patients.
c. The evidence remains unclear about the clinician's reported outcomes.
d. Additional studies should explore how the clinician's learning curve and operational factors affect the applicability and reproducibility of the findings.

## Data Availability

The authors declare that all data generated or analyzed during this study are included in this published article.
